# Analysis of the Bias on the Beidou GEO Multipath Combinations

**DOI:** 10.3390/s16081252

**Published:** 2016-08-08

**Authors:** Yafei Ning, Yunbin Yuan, Yanju Chai, Yong Huang

**Affiliations:** 1 State Key Laboratory of Geodesy and Earth’s Dynamics, Institute of Geodesy and Geophysics, 340 Xudong Rd., Wuhan 430077, China; yybgps@whigg.ac.cn (Y.Y.); cyjigg@whigg.ac.cn (Y.C.); 2 University of Chinese Academy of Sciences, No. 19A Yuquan Road, Beijing 100049, China; 3 School for Engineering of Matter, Transport and Energy, Arizona State University, Tempe, AZ 85282, USA; hy2000@whut.edu.cn

**Keywords:** Beidou GEO, multipath combinations, code bias, correction model

## Abstract

The Beidou navigation satellite system is a very important sensor for positioning in the Asia-Pacific region. The Beidou inclined geosynchronous orbit (IGSO) and medium Earth orbit (MEO) satellites have been analysed in some studies previously conducted by other researchers; this paper seeks to gain more insight regarding the geostationary earth orbit (GEO) satellites. Employing correlation analysis, Fourier transformation and wavelet decomposition, we validate whether there is a systematic bias in their multipath combinations. These biases can be observed clearly in satellites C01, C02 and C04 and have a great correlation with time series instead of elevation, being significantly different from those of the Beidou IGSO and MEO satellites. We propose a correction model to mitigate this bias based on its daily periodicity characteristic. After the model has been applied, the performance of the positioning estimations of the eight stations distributed in the Asia-Pacific region is evaluated and compared. The results show that residuals of multipath series behaves random noise; for the single point positioning (SPP) and precise point positioning (PPP) approaches, the positioning accuracy in the upward direction can be improved by 8 cm and 6 mm, respectively, and by 2 cm and 4 mm, respectively, for the horizontal component.

## 1. Introduction

The Beidou navigation system has been providing position, navigation and timing (PNT) services since 27 December 2012, focusing on the whole Asia-Pacific region. The system constellation comprises 15 launched satellites, out of which 13 (five GEO + five IGSO + three MEO) were fully operational in 2015. It has the ability to transmit signals centred on the B2 (1561.098 MHz), B7 (1207.14 MHz) and B6 (1268.52 MHz) signals.

In recent years, several studies and experiments on Beidou have been conducted, particularly on the precise applications [1,2,3,4,5]. Wuhan University can now provide the daily Beidou precise ephemeris, determined using the BeiDou Experimental Tracking Stations [BETS] data, and the orbit precision of the IGSO and MEO satellites can now reach 10 cm in the radial direction [4]. The comparable accuracies prove that as a GPS system Beidou can be a powerful tool for precise positioning. Particularly in the Asia-Pacific region, Beidou is considered as a relevant and valuable complement to legacy systems for establishing improved GNSS services in this area [2]. Current studies indicate that the real-time horizontal component can reach 5 cm, while the vertical component is within 10 cm after the convergence period [6]. Using the PPP approach to determine zenith tropospheric delay (ZTD), the precision of using Beidou observations alone is comparable with that using GPS only [7]. To make better use of the Beidou system, the Beidou signal characteristics must be fully evaluated.

The multipath delay has been realized as having non-negligible impacts on the quality of observation signals [8,9]. In regard to Beidou, it has been commonly agreed that multipath effects slow down the convergence efficiency and degrade the reliability of positioning resolutions [10,11]. In the recent studies of multipath delay, a systematic code bias that can exceed 1 m has been detected [12,13,14,15,16]; the source of the sensors, which have the appearance of a code multipath, is attributed to the satellite because they are consistently observed using different receivers and antennas [13,14,17]. The magnitude of code bias can be many times larger than the standard deviation of the pseudorange observation noise, and numerical tests demonstrated that the code bias can cause 10 cm deviation of the vertical component in a single-frequency PPP solution [14]. To better understand this bias, the variations of code bias with time and/or elevation were investigated and compared with those of GPS, GLONASS and Galileo. The results showed that the Beidou code bias is not constant over time, but rather has periodic patterns; moreover, the corresponding multipath combinations display systematic variations with elevation, which are significantly different from those of GPS, GLONASS and Galileo [18]. Experiments have also validated that the biases in MEO and IGSO satellites are elevation-dependent and that the systematic shape trends could be eliminated through between-station-differencing or modelling corrections [19]. Cross-correlation suggests that the GEO code bias in MEO and IGSO satellites might vary according to their relative geometries with the Sun and that the bias has a striking day-to-day repeatability [11,17]. Based on these findings, some scholars have extracted the low-frequency component of the first day and employed it as an empirical value to correct the observations correspondingly. Based on an experiment conducted using a local network, results proved that the precision of positioning estimations could be improved after applying such corrections in the relative positioning approach [20]. A similar experiment was conducted at two stations in Perth (Australia) which proved that the precision of single point positioning could also be improved after such corrections are applied [11]. To better analyse and handle the code bias, the algorithms of wavelet transformation and sidereal filtering are also used as tools to make empirical models for some certain stations or satellites [11,17].

As we all know, it is of great relevance to study the code bias in GEO satellites to improve the Beidou positioning accuracy. According to the above discussions, it seems that there are three issues that need to be further addressed. Firstly, the observation data used in the previous experiments are from the year 2012 to 2013, which may be not applicable to the current Beidou in orbiting satellites due to the Beidou constellation having evolved afterwards, thereby necessitating the use of more and newer data to further verify the reliability of some previous conclusions. Secondly, nearly all the relevant experiments were conducted in a local region, meaning we cannot verify whether the GEO code bias is free from azimuth and cannot evaluate the effects of the code bias on the positioning precision in the entire Beidou coverage area. Finally, the corrections regarding the GEO code bias are not properly modelled in a quantitative manner. In fact, it will become more convenient to mitigate this bias if some quantitative and general correction models are proposed.

This paper aims to gain more insight into the GEO-based multipath bias and is organized as follows: in Section 2, the fundamentals of detecting pseudorange bias are introduced. In Section 3, the Beidou GEO pseudorange bias is investigated and compared with that of GPS, Galileo and Beidou MEO satellites. Some mathematical algorithms, such as Spearman correlations, Fourier transformation and wavelet decomposition, are employed to identify the correlations between code bias and elevation, as well as the bias and time series. After this, an empirical model to handle this code bias is also proposed. To evaluate the performance of the correction model, some validation experiments are given in Section 4. Finally, some conclusions are given.

## 2. The Fundamentals of Detecting the Code Bias

The code bias has the appearance of multipath delay, which is always hidden in the multipath series. To detect the code bias, we should obtain the corresponding multipath series first. A so-called multipath combination (*MP*) is often employed to estimate the multipath delay; the *MP* delay on frequency *i* can be expressed as:

(1) $$MP_{i}=P_{i}+(\eta_{\left(i,j,k\right)}-1)\cdot \lambda_{j}\cdot \varphi_{j}-\eta_{\left(i,j,k\right)}\cdot \lambda_{k}\cdot \varphi_{k}+B$$

where *P* and *ϕ* denote the code and phase measurements, respectively; *λ* is the wavelength; *i, j, k* are used to denote different frequencies. The linear factor *η*_(*i,j,k*)_ is calculated as:

(2) $$\eta_{\left(i,j,k\right)}=\frac{\lambda_{i}^{2}+\lambda_{j}^{2}}{\lambda_{j}^{2}-\lambda_{k}^{2}}$$

The linear factor is selected in such a way that the ionospheric delay and non-dispersive delays are cancelled out, leaving the ambiguity differences between the different phase measurements lumped together in *B*, which is determined as average over raw *MP* values for each continuous ambiguity block. As a consequence, no absolute *MP* values are known, just the *MP* variations over continuous ambiguity blocks. However, long time changes in the pseudorange observations can still be found in the *MP* time series, which is the basis of our detecting code bias. In most practical applications, setting *i* = *j* or *i* = *k* can make the computation more convenient.

The theoretical maximum multipath effect on carrier phase is only one quarter of the carrier wave cycle. For Beidou, the maximum multipath effects for B2, B7 and B6 are 4.8, 6.2 and 5.9 cm, respectively. With respect to the magnitude of code bias that is at the level of decimetres or even metres, the multipath effects on the carrier phase are negligible.

## 3. The Analysis of the Geo Satellite-Based Code Bias

The multipath bias can be comprehensively analysed using different mathematical tools. Spearman correlation, Fourier transformation and wavelet analyses are all employed in this section. The Spearman correlation coefficient is a distribution-free rank statistic for evaluating the strength of the monotone association between two independent variables. Comparing with other correlation approaches, e.g., Pearson analysis, the Spearman correlation, which operates on the ranks of the data rather than the raw data, does not require a linear relationship between variables. Moreover, Spearman correlation estimation is insensitive to extreme data, which is an advantage because the measurement errors in the pseudorange observables will be neglected by this algorithm [21]. In Section 3.1, we use the Spearman correlation-analysis to determine the correlations between the code bias in Beidou GEO satellites and elevation, as well as code bias and time series. The Fourier transformation is able to depict the multipath time series through amplitude versus frequency, revealing the frequency and the amplitude of each component [22]. In Section 3.2, we take the multipath series as a discrete signal and use the Fourier transformation to investigate the spectral characteristic of multipath time series in the frequency domain, which helps to further identify the periodicity characteristic of the code bias hidden in the multipath series. Wavelet analysis allows us to decompose complicated information contained in a signal into elementary functions associated with different time scales and different frequencies and to reconstruct it with high precision and efficiency [23]. In Section 3.3, wavelet transformation is utilized to isolate the signal with daily periodicity from those at other frequencies; after that, we use the least squares method to estimate the model unknowns. The following sections will explicitly describe the three mentioned approaches.

### 3.1. The Detection of the GEO Satellite-Based Code Bias

#### 3.1.1. The Comparison of GEO-Based Code Bias with Other Systems

To better understand the characteristic of the Beidou GEO code bias, we compare the multipath effects induced from GEO satellites with those from GPS and Galileo systems. To make the comparison fair, the Beidou MEO satellites are also selected because all the satellites in the other two navigation systems considered are both MEO satellites.

**Figure 1:**
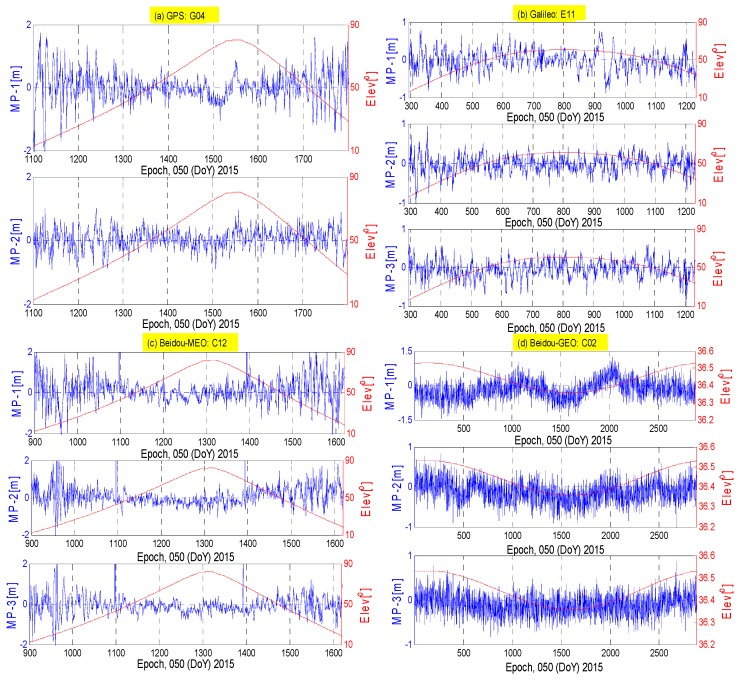
The multipath estimations (**blue**) and satellite elevation (**red**) for (**a**) GPS satellite G04; (**b**) Galileo satellite E11; (**c**) Beidou MEO C12 and (**d**) GEO C02 at station CUT0. Each row represents the multipath estimations for different frequencies.

Figure 1 illustrates the multipath series of satellites G04, E11, C12 and C02 and summarizes the correlations between the multipath estimation and the elevation, as well as the time series. From Figure 1a–c, it can be observed that the amplitude and dispersion of the code multipath of the MEO satellites increase significantly as the elevation decreases. The multipath effect nearly increases to 2 m on the first frequency for the GPS system when the elevation is below 20° and the first frequency observable is more sensitive to the multipath effect than the second frequency. The code multipath of the Galileo satellite has the smallest amplitude and dispersion, for which the multipath variations are almost below 0.5 m and seem independent of elevation. Compared with (a) and (b), the multipath estimation of the Beidou MEO satellite (c) has a trough in all the time series when the satellite has the maximum elevation, and the amplitude becomes bigger when the elevation decreases, which suggests that there is an elevation-dependent bias in the multipath delay. In regard to the Beidou GEO satellite (d), the magnitude of multipath variation is many times bigger than that of the elevation variation, and the trend of multipath series behaves somewhat systematically correlated with time series, which are evidently different from the Beidou MEO satellites. These results agree well with some mentioned references.

The code multipath estimations on different frequencies of GPS, Galileo and Beidou satellites show almost the same pattern, while the pattern of the counterparts of GEO satellite from the first frequency has a little deviation with respect to those from the second and the third frequencies. It suggests that the GEO code biases which are absorbed in the multipath delay of the three frequencies need discriminative treatments, taking their different behaviors into account.

**Figure 2:**
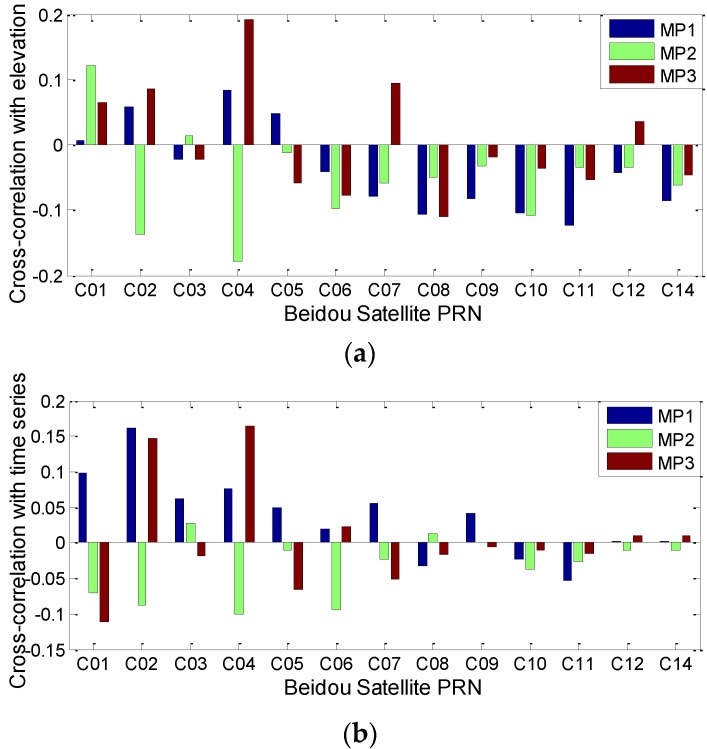
The Spearman cross-correlations between multipath estimations with elevation (**a**) and time series (**b**) at station CUT0.

**Figure 3:**
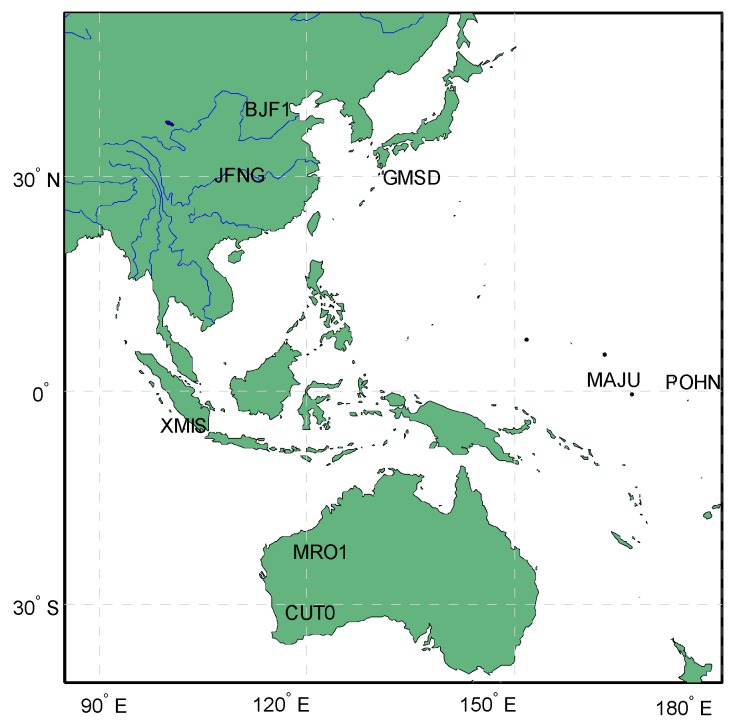
The distribution of the eight experimental stations.

Figure 2 shows the Spearman cross-correlations between the multipath delay and the elevation, as well as time series. For Beidou non-MEO satellites, nearly all the correlations with elevation are negatively related, except those for C07 and C12 on the third frequency, and the correlations between multipath delay and time series are quite low and only partially positive, considering different satellites and frequencies. These results, to some extent, verify the existence of elevation-dependent code bias in the multipath delay. For Beidou GEO satellites, the correlation results show that the multipath delay has correlations with both time series and elevations. However, the GEO satellites show daily variations in their fixed frames of the order of approximately 2000 km, which results in their elevation also having the daily variation periodicity characteristic [11]. The variation of GEO code bias should be carefully analysed to be reasonably mitigated. For this purpose, it becomes necessary to analyse observations from some stations with different elevations. Although some conclusions have been made before [11,17], to guarantee the correctness and reliability of the following findings, we conduct this work again by using newer and more data collected from stations that are distributed over the entire Asia-Pacific region. Based on this, we select 8 stations distributed over the Asia-pacific region as experiment subjects; their distributions are depicted in Figure 3. The observation sampling interval is 30 s. Most of the stations come from the MGEX network, except station BJF1, which comes from the Chinese iGMAS network. To explicitly describe the periodical variation trends of the GEO-based code bias over consecutive days, we select the multipath series from 50 to 55 days of year (Doy), 2015, as representative results to illustrate the multipath effects.

The code observations of the first frequency have the best signal quality, being able to clearly and directly present the required results [1,19]. On the contrary, many unrelated high-frequency signals are included in the third frequency observations; the following result (i.e., Table 1) also suggests that the amplitude of periodical variations of the third frequency is the smallest. The performance of the code observations of the second frequency is in between those of the other two frequencies. To show the periodicity characteristic of the code bias, as well as present how to detect and remove unrelated signals, we set the observations of the second frequency as an example to conduct the relevant analysis in Section 3. For simplicity sake, the multipath results of the other frequencies are not displayed here.

#### 3.1.2. The Comparison of GEO Code Bias in Satellites C01/C02/C04 and C03/C05

Many primary investigations have verified that the satellite-induced code bias is independent of receiver types and free from observation environments [14,18,20]. Moreover, Zhao et al. found that the GEO phase bias, which varied with satellite elevation at the Northern Hemisphere stations, had a trend opposite to that in the Southern Hemisphere [17]. Based on this finding, the pseudorange bias may also have a similar characteristic. Thus, in the following analyses, we select stations JFNG and CUT0, which, respectively represent stations in the Northern and the Southern Hemisphere, to investigate the effects of azimuth and elevation on the code bias.

**Figure 4:**
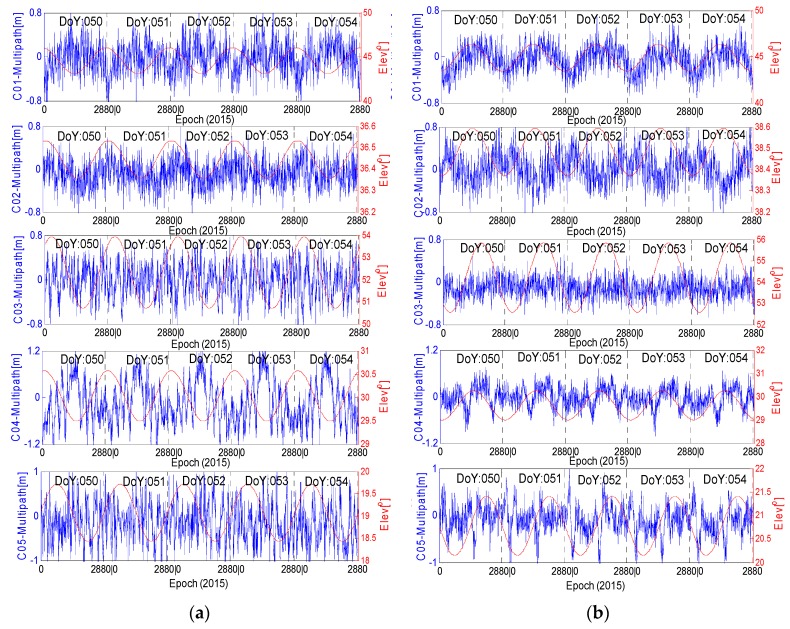
The relationship between MP series and elevations for GEO satellites at station CUT0 (**a**) and JFNG (**b**). Blue represents the multipath series; red represents the elevations. The observation sampling rate is 30 s.

The multipath estimations of the two stations are presented in Figure 4; the figure shows two phenomena: (1) the elevation variation of the GEO satellites at station JFNG is opposite to that at station CUT0; (2) the variation trends of the GEO multipath series are nearly the same at the two stations. The GEO satellites move around the Equator. When they move closer to one station, the elevation will decrease for the station in the opposite hemisphere, which is the reason for the first phenomenon. The second phenomenon shows that the code bias is independent of the azimuth and the elevation and that the systematic multipath delay is most related to the satellite due to the two stations having obviously different observation environments. Based on these two phenomena, the code bias varying with satellite elevation in the Northern Hemisphere has a trend opposite to that in the Southern Hemisphere, which agrees well with the experimental results regarding the phase bias [19].

**Figure 5:**
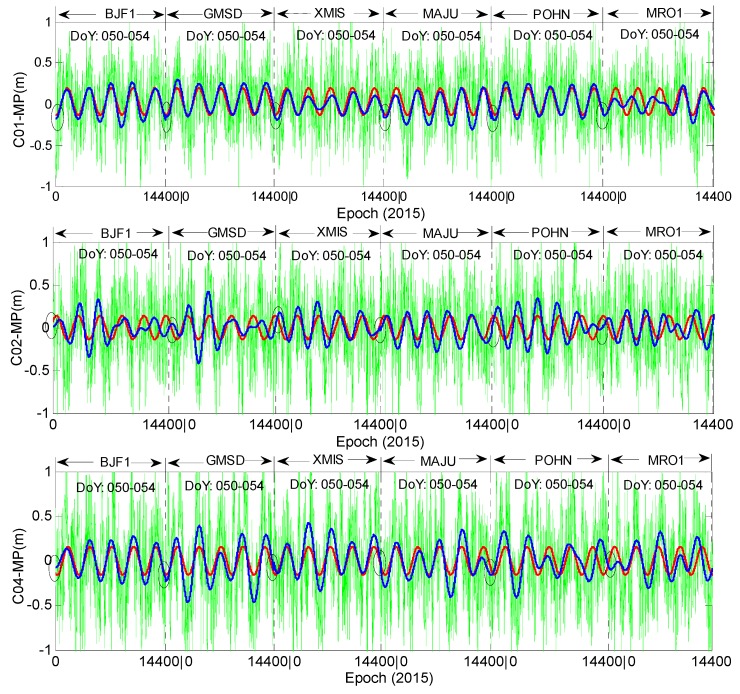
The multipath series of C01, C02 and C04 at the other six stations. Green is the multipath estimation; red is the Fourier fitting results of the multipath series; blue is the wavelet reconstructed multipath series; the cycle is the edge of reconstructed multipath series between different stations. The observation sampling rate is 30 s.

**Figure 6:**
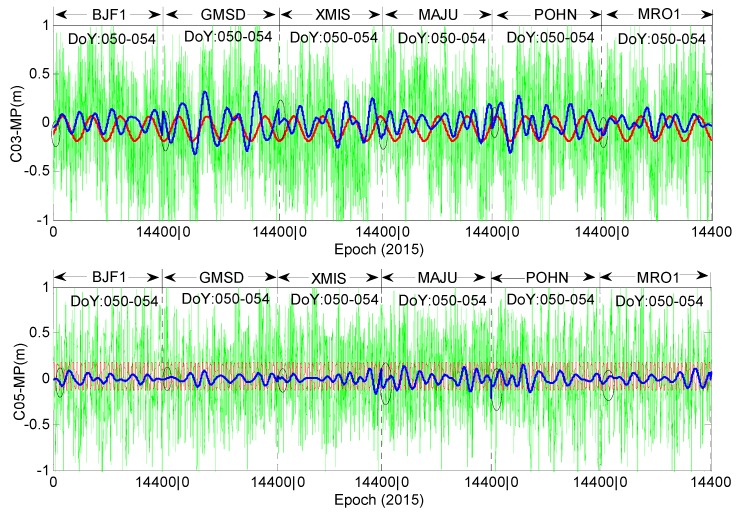
The multipath series of C03 and C05 at the other six stations. Green is the multipath estimation; red is the Fourier fitting results of the multipath series; blue is the wavelet reconstructed multipath series; the cycle is the edge of reconstructed multipath series between different stations.

From the results in Figure 4 and Figure 5, evident daily periodicity can be observed in the multipath time series of C01, C02 and C04. Their initial values and variation trends are similar across the three satellites, further indicating that the time-dependent bias is most related to the satellite instead of the elevation or station. Take stations CUTO and JFNG as an example; these two stations are distantly separated and their observation environments are independent of each other; their elevation trends are even opposite, while the trends of the multipath estimations at the two stations are much similar for each satellite of the three. In this sense, the multipath delays from C01, C02 and C04 might not only contain effects from the surroundings of the station but also contain some bias from the satellite, e.g., internal hardware delay [19]. In Figure 4, the trend of C04 at station CUTO has an obvious characteristic of periodicity variation, but this is not the same for station JFNG; the reason for this phenomenon will be discussed in the following sections. In Figure 5, for the three GEO satellites (C01,C02 and C04), the shape of each multipath series at the 6 stations seems sinusoidal, the shape of each multipath series at the experimental stations seems sinusoidal, which has also been demonstrated in reference [11], and their maximum variation ranges are all limited to 0.12 m to 0.16 m. In this sense, the multipath biases at the 6 stations seem to have similar variation shapes. Figure 6 shows that the variations of C03 and C05 illustrate the random distribution characteristic, which is evidently different from that in the other three GEO satellites.

**Figure 7:**
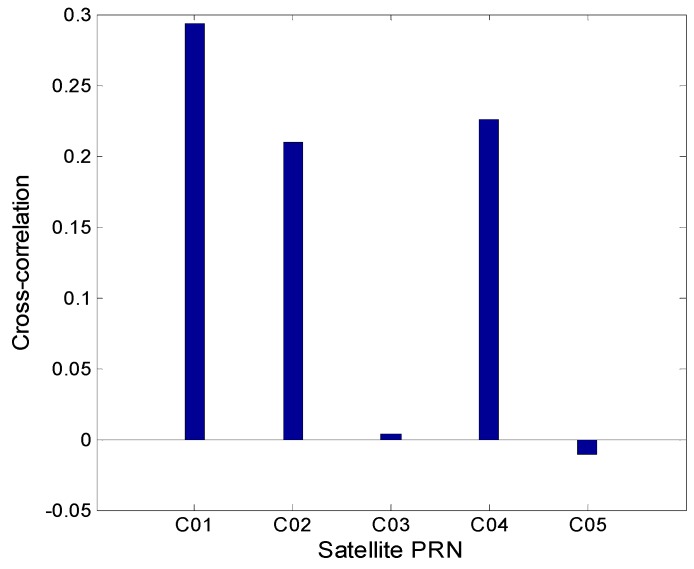
The Spearman cross-correlations between the multipath delay in CUTO and that in JFNG.

Figure 7 presents the Spearman cross-correlations between the multipath estimations at station CUT0 and those at station JFNG. The magnitudes of the correlations of C01, C02 and C04 are many times bigger than those of C03 and C05. This phenomenon verifies that there are some biases in satellitesC01 and C02, which affect the pseudorange observations at the two stations in terms of communal errors. C03 and C05 do not show such systematic bias. Because the data of satellite hardware is not available, the possible reason may be that satellites C03 and C05 do have not such bias or these biases are constants, which are absorbed in *B* in Equation (1). Reference [1] reported that satellite C03 had the best code data quality, with the smallest standard deviation compared with that of C01, C02 and C04 (C05 was not available in that paper); the reason may just be that C03 has no such systematic code bias. The overall magnitudes of all the cross-correlations are not as large as expected, being mainly affected by the code observation noise, whose standard deviations are even larger than the variations of the code bias under some conditions.

**Figure 8:**
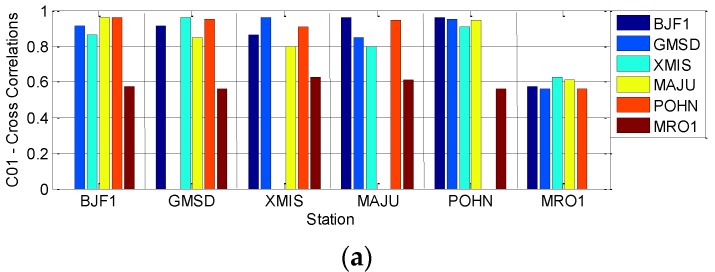

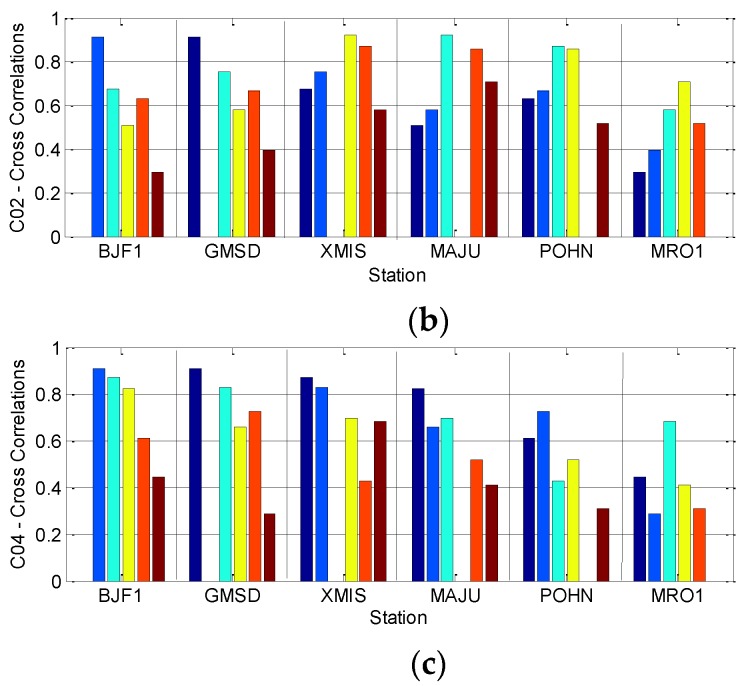
The Spearman cross-correlations between multipath biases at different stations for satellites C01 (**a**); C02 (**b**) and C04 (**c**).

**Figure 9:**
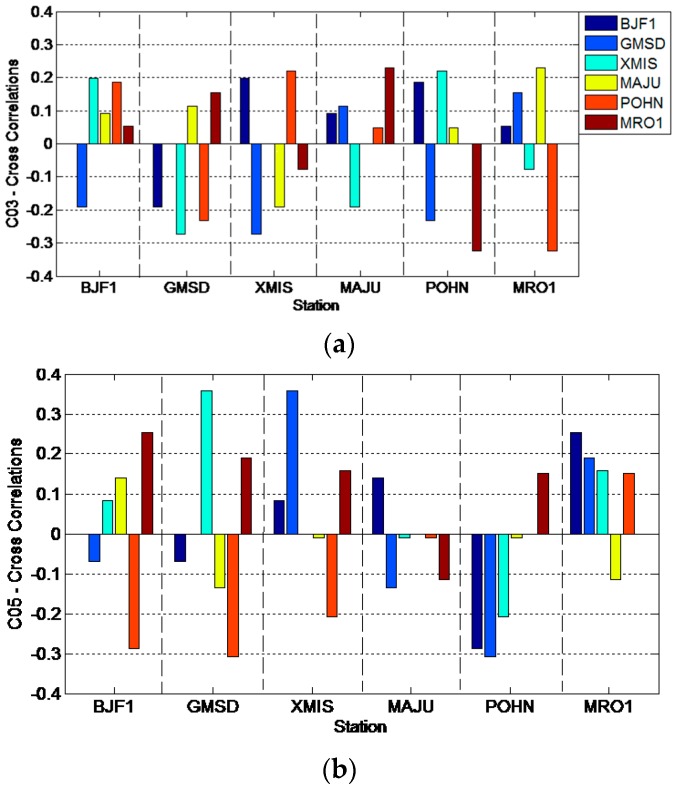
The Spearman cross-correlations between multipath biases at different stations for satellites C03 (**a**) and C05 (**b**).

To quantitatively verify that the biases at the 6 stations are similar for certain satellites, Figure 8 and Figure 9 demonstrate the Spearman cross-correlations between multipath bias at the six stations for all five GEO satellites. Figure 8 presents the multipath correlations for satellites C01, C02 and C04. Here, all the Spearman correlations between multipath series at different stations are positively related. Most of their correlation values are larger than 0.5, except the results of station MRO1, which range from 0.3 to 0.5. These results demonstrate that their multipath biases are highly correlated, which means that the multipath variation trends at the 6 stations will be quite similar; otherwise their correlations will not be positive with such large values. On the contrary, Figure 9 shows that the multipath correlations of C03 or C05 are only partially positive; most correlation values are less than 0.3, which means that the bias variations at the six stations are not similar for satellites C03 and C05. These deductions from Figure 8 and Figure 9 further verify that bias with the daily periodicity characteristic is induced for satellites C01, C02 and C04, while the bias of satellites C03 and C05 has no such characteristic.

### 3.2. The Spectrum Analysis of GEO Code Bias

**Figure 10:**
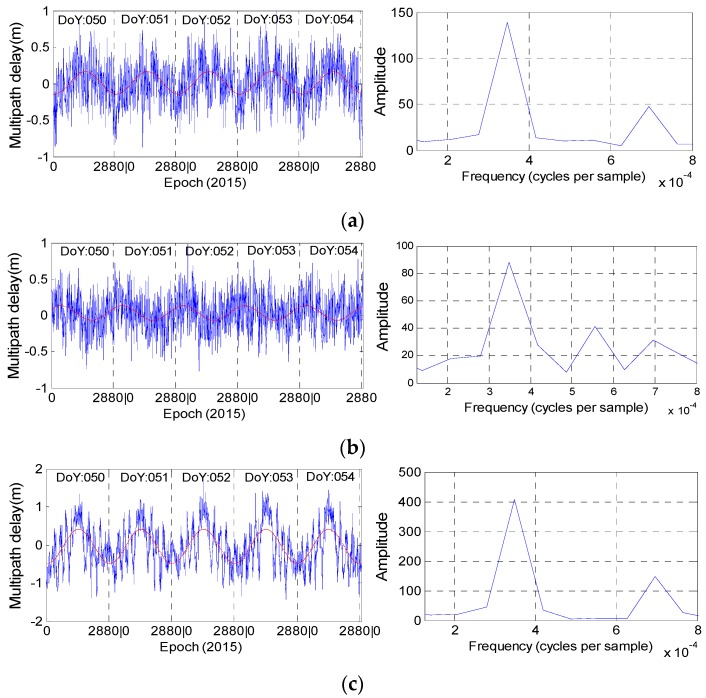
The fitted curve (**red line**) and its corresponding magnitude spectrum (**right**) for C01 (**a**); C02 (**b**) and C04 (**c**) at station CUT0.

**Figure 11:**
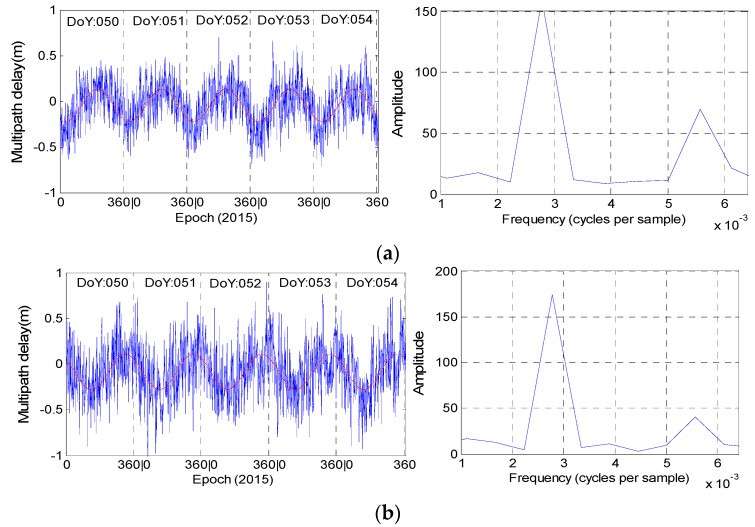

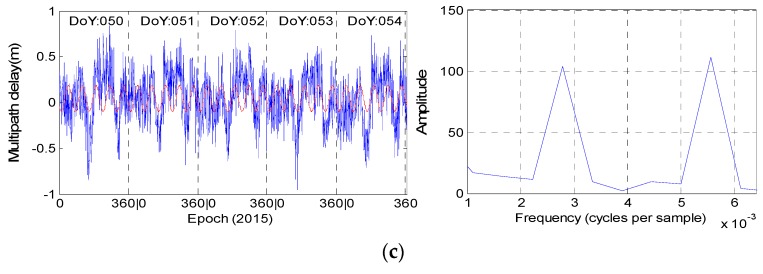
The fitted curve (**red line**) and its corresponding magnitude spectrum (**right**) for C01 (**a**); C02 (**b**) and C04 (**c**) at station JFNG. The observation sampling rate is 240 s.

**Figure 12:**
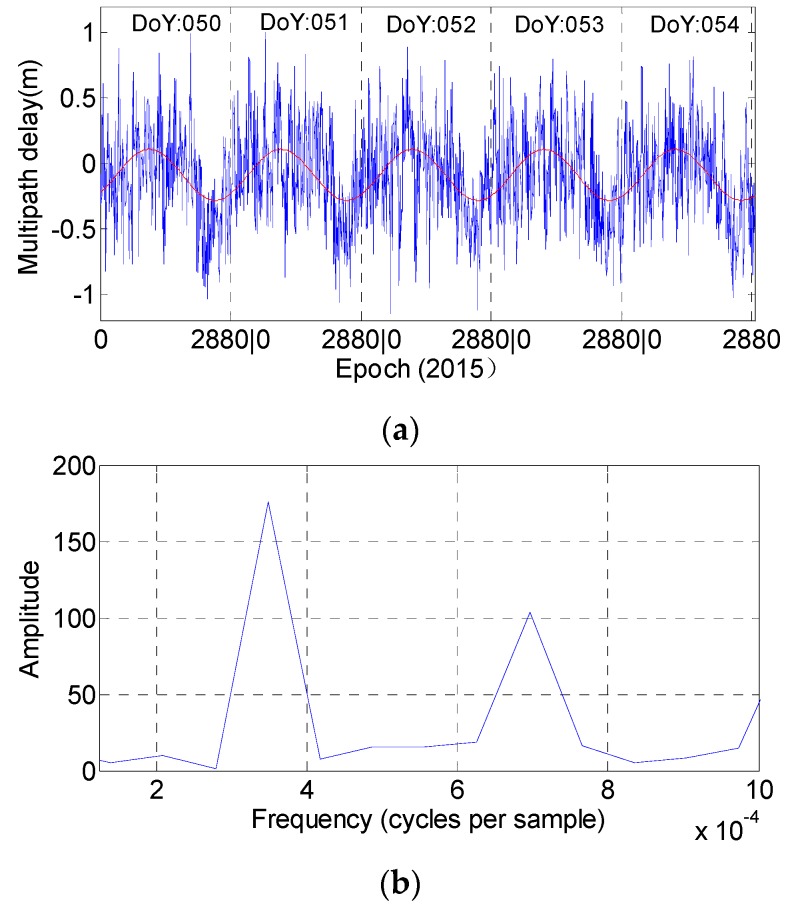
The fitted curve (**red line**) and magnitude spectrum (**b**) of C04 at station GMSD. The observation sampling rate is 30 s. (**a**) Represent the multipath delay during the five consecutive days.

Figure 10 and Figure 11 illustrate the multipath results of stations CUT0 and JFNG, as well as their corresponding fitting curves and magnitude spectrums derived from Fourier transformation. Since we will take the observations at station JFNG as example to illustrate how to use wavelet-reconstruction to reconstruct the raw signal, we resample the data at station JFNG with the interval of 240 s for convenient computation. The sampling interval at the other stations is 30 s. In Figure 10, the fitting curve and magnitude spectrum both illustrate the code bias of C01, C02 and C04 have evident daily periodicity characteristic and similar variation trends. The same holds for station JFNG in Figure 4, except satellite C04. The bottom panel in Figure 11 illustrates there is an additional semi-day signal also included in the multipath delays of C04. However, during the same experiment time span, other stations e.g., station GMSD (Figure 12) illustrates the characteristic of daily periodicity, which reveals an excellent agreement with the results about CUT0. It can be deducted that the semi-day signal of C04 come or derived from its own observation environment, and it is most related to satellite.

**Figure 13:**
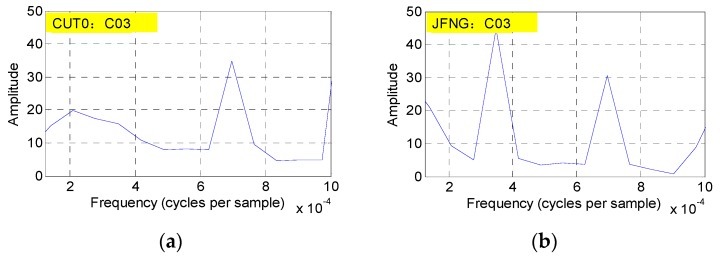

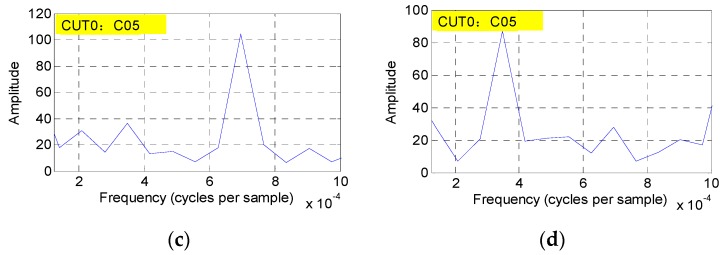
The magnitude spectrum of satellites C03 (**a**, **b**) and C05 (**c**, **d**) at stations CUT0 (**a**, **c**) and JFNG (**b**, **d**).

In practical application, a series can be reliably believed to have the periodicity characteristic when its corresponding amplitude is more than 80. However, Figure 13 shows that the multipath estimations of C03 and C05 could not satisfy this condition at the two stations at the same time, to a great extent, which further suggests that there is no periodicity bias in satellites C03 and C05.

The above results show that C01, C02 and C04 mostly have satellite-induced code bias with a clear periodicity variation characteristic. Satellites C03 and C05 have no such bias; perhaps the magnitude of such bias is too small and can be ignored or their systematic bias is constant, being absorbed by term *B* in Equation (1).

Assume the sampling interval as Δ (ins), frequency as *f* and periodicity as *T*. For the multipath series at stations CUT0 and GMSD, the Δ is 30 s, while *f*, which corresponds to the first peak (also the most evident peak), is approximately 3.47 × 10^−4^ (Hz). For the multipath series at stations JFNG, the Δ is 240 s, while *f* is approximately 2.78 × 10^−3^ (Hz). Using Equation (3), all the periodicities of the multipath combinations of satellites C01, C02 and C04 are nearly equal to 86,400 s, which further quantitatively verifies that the periodicity of their corresponding multipath bias is one day:

(3) $$T=\frac{\Delta }{f}$$

Moreover, a semi-day signal could be detected in all the mentioned magnitude spectrums. Although their corresponding amplitudes are much smaller than those of the signals with the daily periodicity characteristic, such semi-day signal is nearly observable in all the experimental stations. Because the data of the satellite internal hardware is not available, we can only assume that the semi-day signal is most related to satellites.

### 3.3. Time Series-Depending Model for GEO Code Bias

Based on the above analysis, the daily repeating code bias plays an important role in satellites C01, C02 and C04. It is necessary and significant to model this time-dependent bias to mitigate its effect on the positioning. The Beidou observations collected from 040 to 060 (Doy), 2015, at eight stations are used; we resample the data at the interval of 240 s for convenient computation. The computation process can be concluded as follows:

(1) Classify all the observables according to the types of frequency and satellite; after that, we use the wavelet transformation to remove the signal trends;
(2) There have many unrelated high-frequency signals in the raw observations, which disturb the analysis of the special signals that have the daily periodicity characteristic. Considering this, we use wavelet-reconstruction to reconstruct the raw signals. Because the sampling interval (Δ) of the experimental data is 240 s, we use the Daubechies wavelet with a 45 degree vanishing moment to analyse and construct the multipath series. Thus, we obtain the signal with a frequency band of $2^{8}\Delta \sim 2^{9}\Delta$, i.e., 17.06~34.12 h.

**Figure 14:**
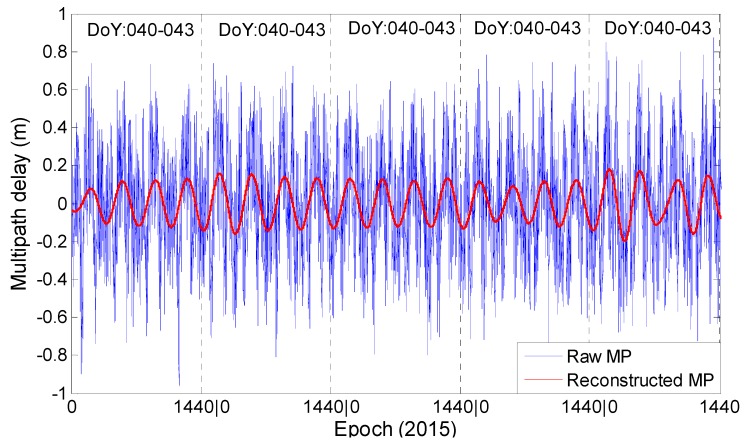
The raw and the reconstructed multipath series of C04 at station JFNG.

After step 2, the reconstructed signal can clearly illustrate the characteristic of daily periodicity. For example, the multipath series of C04 at station JFNG does not illustrate an obvious daily periodicity characteristic in Figure 4 due to a semi-daily signal being included, which is also mentioned in Figure 11. After the second step, Figure 14 shows that the daily periodicity characteristic can clearly be observed in the reconstructed signals.

(3) After many fitting methods were tested, we found that the trigonometric function is the most effective for this study because this method yields the smallest RMS value among the tested fitting approaches. The estimation of code bias *Cb* can be modelled as:
  (4) Cb=a⋅cos(x⋅ω)+b⋅sin(x⋅ω)
  where *x* represents the time difference between observation time and midnight on 050 (Doy), 2015, with the unit of seconds, and *ω*, *a*, *b* are the unknown parameters. To facilitate the least squares adjustment, we transform Equation (4) into the standard indirect adjustment model; it is expressed as:
  (5) $$v=-\sin(x\cdot \overset{o}{\omega })\cdot \Delta a+\cos(x\cdot \overset{o}{\omega })\cdot \Delta b+[-\overset{o}{a}\cdot \sin(x\cdot \overset{o}{\omega })\cdot x+\overset{o}{b}\cdot \cos(x\cdot \overset{o}{\omega })\cdot x]\cdot \Delta \omega -\overset{o}{Cb}$$
  where $\overset{o}{\omega },\;\overset{o}{a},\;\overset{o}{b}$ are the corresponding prior-estimations of the three unknowns and $\overset{o}{Cb}$ is the value of the reconstructed signal at time *x*. After the least squares adjustment of the model unknowns, all the unknown estimates are summarized in Table 1. The results indicate that the magnitudes of bias corrections of the first and second frequencies are at the same level, both being bigger than that of the third frequency. The periodicity signal, which is extracted from the multipath series of the third frequency, has the lowest amplitude. We use the equation:
  (6) $$T=\frac{2\cdot \pi }{\omega }$$
  to estimate periodicity *T*. The results show that periodicity varies from 86,069 to 86,237 s for different frequencies and satellites, being roughly equal to the length of one sidereal day, i.e., 86,160 s. Note that the estimations of *ω*, *a*, *b* will change with the time varies; details of the relevant deductions will be discussed in another paper.

## 4. Experiments

Turning now to the evaluation of the performance of the GEO code bias model, single point positioning (SPP) and precise point positioning (PPP), with 20 days of Beidou observations, are conducted and the results are compared. We mainly describe the results at station CUT0 for simplicity. During the experiments, the precise station coordinates come from weekly solutions, and positioning errors can be computed by subtracting the estimated coordinates from the precisely estimated coordinates [24,25].

**Figure 15:**
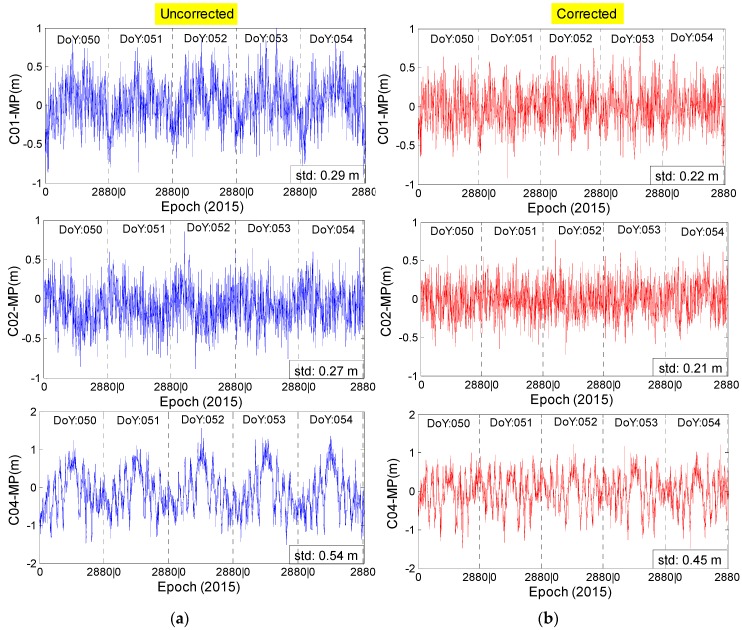
The multipath estimations of the second frequency without (**a**) and with (**b**) corrections for code bias. “std” represents the standard deviations of multipath series.

For comparison with the multipath series of satellites C01, C02 and C04 in Figure 4, the right panel in Figure 15 depicts the multipath estimations to which the time-dependent correction model has been applied. After applying the model, the time-dependent periodicity characteristic in the previous multipath estimations almost disappears. The standard deviation (std) decreases from 0.29 m to 0.22 m for satellite C01 and decreases by 0.06 m and 0.09 m for C02 and C04, respectively. It can be deduced that the signal quality is improved after the model has been applied.

**Figure 16:**
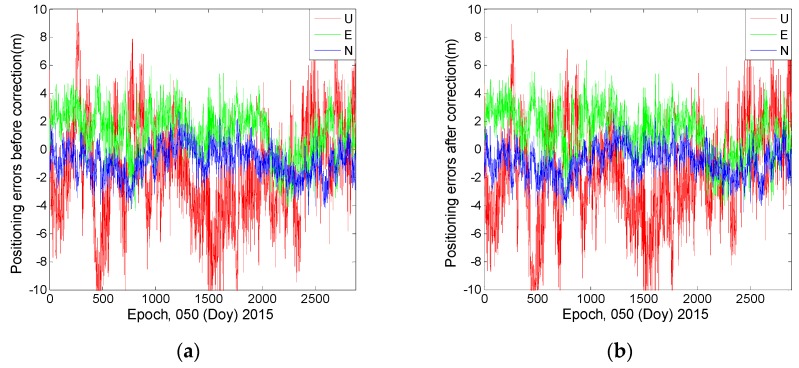
The SPP results estimated by using ionosphere-free combination of the first and second frequencies at station CUTO. (**a**) Represents the positioning errors before applying the correction model; (**b**) Represents the positioning errors after applying the correction model.

**Figure 17:**
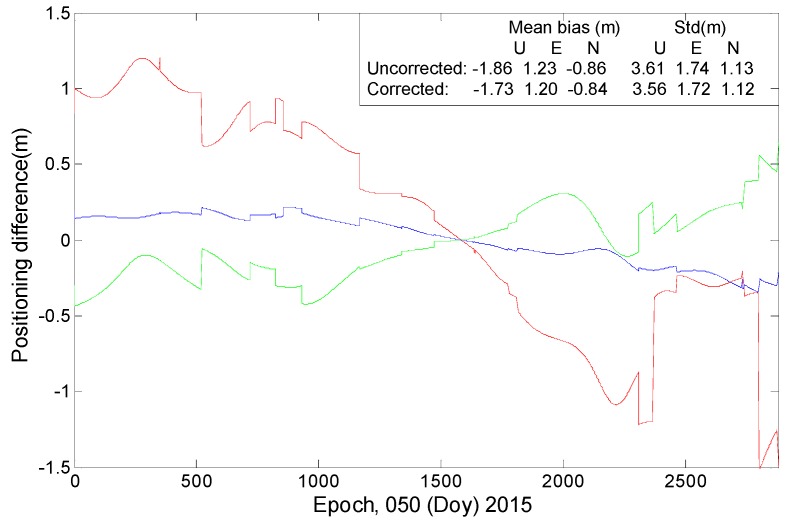
“Positioning difference” is the difference between SPP estimations with and without employing the correction model. The “mean bias” represents the equal result of the positioning errors. “Uncorrected” indicates the results when the code bias is not corrected; “Corrected” indicates the results after the model has been applied.

SPP, which uses code measurements to produce coordinates, is usually affected by the code bias. Figure 16 and Figure 17 show that the correction model has certain effects on the SPP estimations. After the model is applied, the positioning bias decreases by 0.13 m in the U direction and by approximately 0.03 m in the horizontal components. A decrease of more than 0.05 m is also found in terms of the standard deviation in the upward (U) direction. The correction model performs better regarding the improvement of position precision in the U direction rather than in the horizontal components.

**Figure 18:**
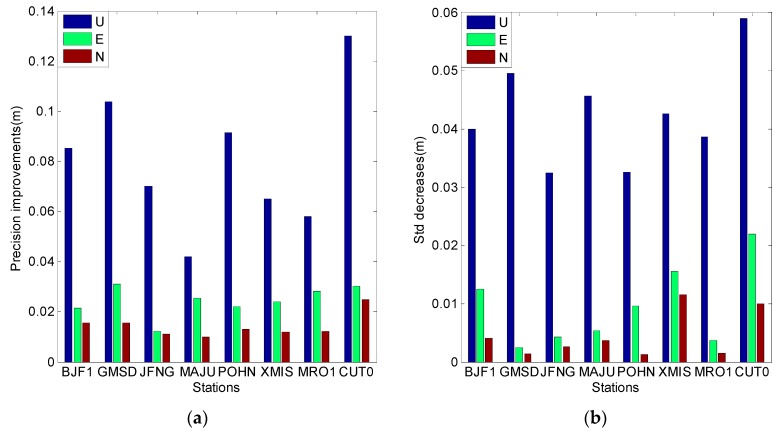
The SPP results estimated by using ionosphere-free combination of the first and second frequencies. (**a**) Describe precision improvements after model correction; (**b**) Describes the Std decreases after the model correction.

**Figure 19:**
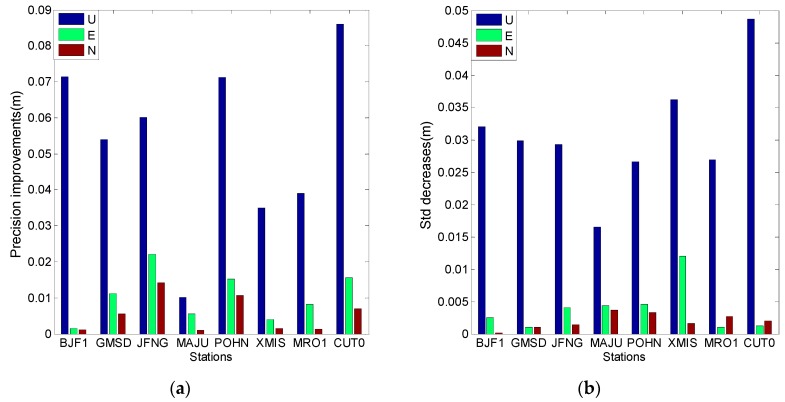
The SPP results estimated by using ionosphere-free combination of the first and third frequencies (**a**) describes the precision improvements after model correction; (**b**) Describes the Std decreases after the model correction.

Figure 18 summarizes the average results of the 20 days for each experimental station; it is based on ionosphere-free combination of the first and second frequencies. The improvement of positioning precision in the U direction is the highest, and the precision improvement in the east (E) direction is lower than that in the U direction. The precision improvement in the north (N) direction is the lowest, which may be caused by the geometric structure of the three corrected GEO satellites. The overall correction values in Figure 19 are smaller than those in Figure 18, possibly because the correction value of the third frequency is smaller than that of the second frequency.

**Figure 20:**
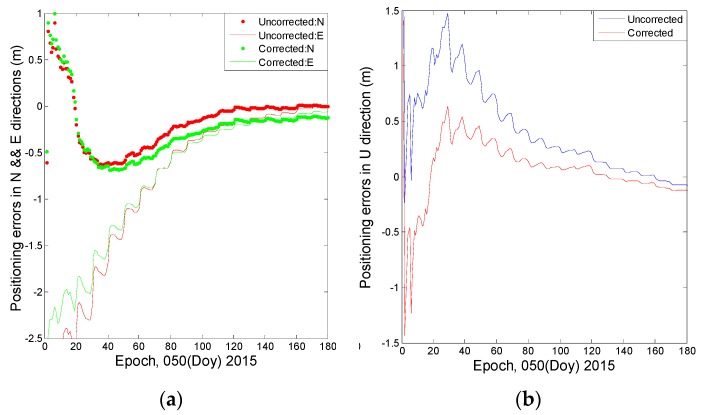
The positioning errors during the first 180 epochs at station CUT0based on the corrected and raw observations. (**a**) Describes the positioning errors in N and E directions; (**b**) Describes the positioning errors in U direction.

The pseudorange measurements occupy 1/10000 weights in the static PPP approach with respect to the phase observables, which makes the pseudorange observations contribute marginally to the final positioning precision [7]. However, the pseudorange has a great effect on the convergence efficiency; Figure 20 illustrates the positioning errors during the first 180 epochs at station CUTO. After converging with 80 epochs, the positioning precision, which is based on corrected pseudorange observations, improves by 0.16 m, 0.08 m and 0.01 m with respect to that using the raw observables in the U, E and N directions, respectively.

**Figure 21:**
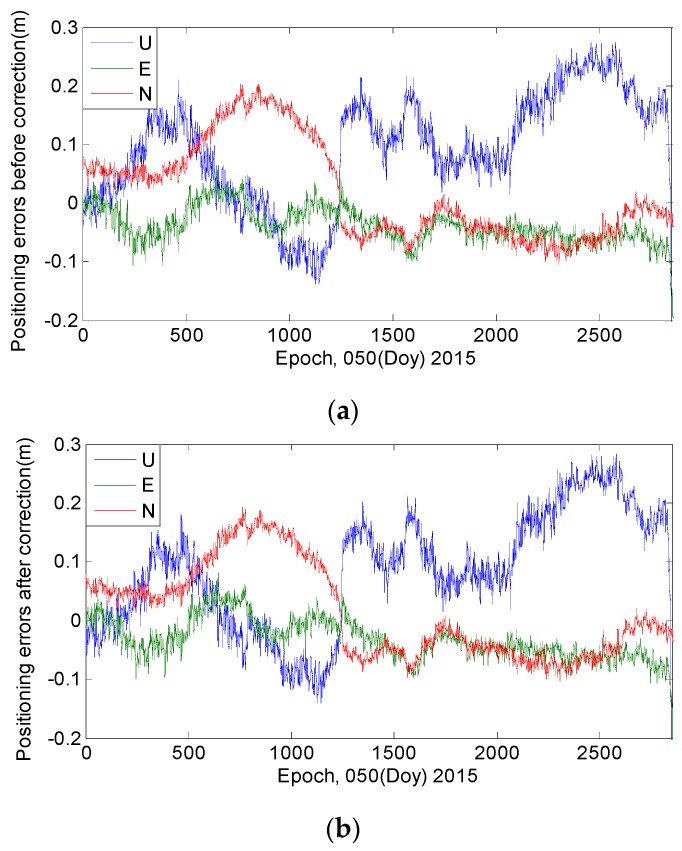
The position estimations of the kinematic PPP approach by using ionosphere-free combinations of the first and the second frequencies. (**a**) Represents the positioning errors before applying the correction model; (**b**) Represents the positioning errors after applying the correction model.

**Figure 22:**
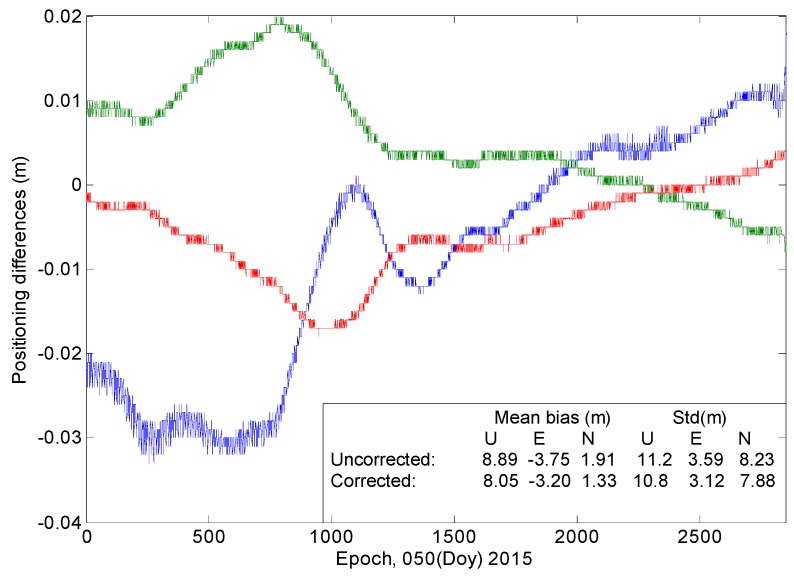
“Positioning difference” is the differences between kinematic PPP estimations with and without employing the correction model. “Uncorrected” indicates the results when the code bias is not corrected; “Corrected” indicates the results after the model has been applied.

Figure 21 and Figure 22 illustrate that the correction model also affects the kinematic PPP estimations. We estimate the positioning results by using the kinematic PPP approach with a loose constraint of 1 m to positions at two adjacent epochs. It is interesting to find that the differences (bottom panel) are almost opposite to the positioning errors (up), as for the SPP experiment (Figure 16).The positioning precision at station CUT0 improves by nearly 0.9 cm in the vertical direction and by 0.7 cm in the horizontal component after the correction model is applied.

**Figure 23:**
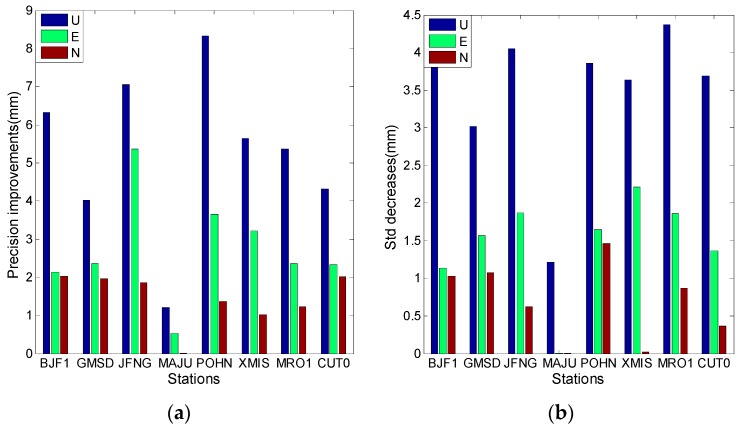
The kinematic PPP results using ionosphere-free combination of the first and second frequencies. (**a**) Describes the precision improvements after the model is applied; (**b**) Describes the Std decreases after the model is applied.

**Figure 24:**
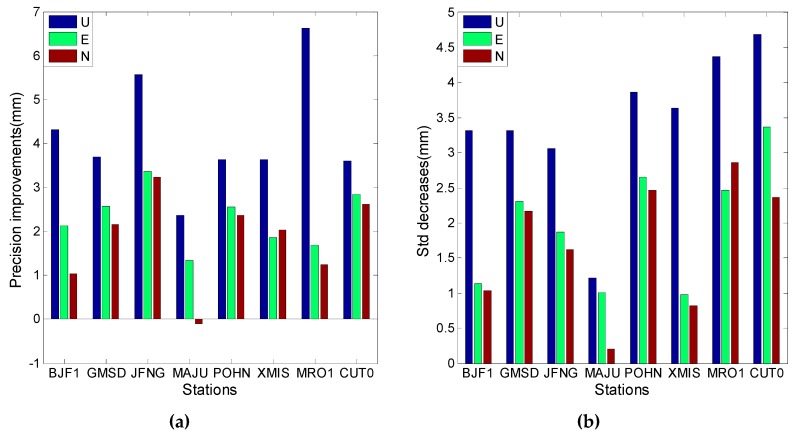
The kinematic PPP results using ionosphere-free combination of the first and third frequencies. (**a**) Describes the precision improvements after the model is applied; (**b**) Describes the std decreases after the model is applied.

Figure 23 and Figure 24 illustrate the performance of the model in the kinematic PPP approach. The model has a better performance for the combination of the first and second frequencies than for the combination of the first and third frequencies. The improvements of positioning precision in the U direction can be easily observed in the two figures, and the horizontal components have a little deviation and do not seem to have any cross-correlations between the two ionosphere-free combinations. The equal improvements are approximately 6 mm in the U direction and 3 mm for the horizontal components. However, Figure 24 shows one exception, i.e., the model decreases the positioning precision in the N direction at station MAJU. One of the possible explanations can be corrections of the first frequency are not totally in agreement with the truth; the corrections of the second frequency could make up for this shortcoming, while the corrections of the third frequency cannot do so due to values with smaller magnitude. This suggests that these exceptions mainly occur for the combinations of the first and third frequencies and not for the combinations of the first and second frequencies.

## 5. Conclusions

In this paper, the most recent GNSS observables collected from eight stations in the Asia-Pacific region are employed to investigate the characteristic of code bias of the Beidou GEO satellites. Employing some algorithms, such as Spearman correlation analysis, Fourier transformation and wavelet decomposition, this paper validates that the code multipath combination of the BDS satellites not only contains multipath effects from the surroundings of the stations but also some multipath-like biases from the satellites. The multipath bias shows the daily periodical characteristic as well as similar variation trends of the eight experimental stations for GEO satellites C01, C02 and C04; this was not detected in C03 and C05. Experiments also verify that the periodical variation of the multipath bias on satellites C01, C02 and C04 is most related to the satellites rather than the external observation environments. This finding could help verify the hardware questions for GEO satellites C01/C02/C04 through comparison with C03 and C05. By analysing the spectrum amplitude results, we also detected a semi-day signal in our experiments; this signal is observable in nearly all the multipath series. We think that it is an issue that deserves further analysis.

We present the correction model by extracting the multipath combinations from many previous days. Validation experiments verify, after the model has been applied, that the multipath residuals become random and the periodicity characteristic in the previous multipath series is nearly removed. The convergence efficiency is thus improved in the static PPP experiment. Moreover, the precisions of single point positioning and kinematic precise positioning estimations are also improved, especially in the U direction.

In general, this manuscript particularly researched the Beidou GEO satellites, aiming to reveal more characteristics of the code bias in GEO satellites. Based on our findings, more mathematical models and physical settings are expected to be presented to mitigate the GEO multipath delay, thereby improving the Beidou positioning accuracy.

## Figures and Tables

**Table 1:** The unknown estimations in Equation (3).

PRN	Unknown	MP1	MP2	MP3
C01	w	7.30017 × 10^−5^	7.28874 × 10^−5^	7.28730 × 10^−5^
	a	−0.125328	−0.02646	0.029274
	b	0.125916	−0.151284	0.08595
C02	w	7.28925 × 10^−5^	7.28592 × 10^−5^	7.29035 × 10^−5^
	a	−0.120288	0.080346	0.0979896
	b	0.0146496	0.0322392	0.021256
C04	w	7.28308 × 10^−5^	7.29264 × 10^−5^	7.28806 × 10^−5^
	a	−0.117576	−0.121176	−0.04878
	b	−0.064608	0.0345504	−0.02712
